# Finding balance: the dynamic interplay between H3K27me3 writers and erasers in regulating environmental plasticity and memory

**DOI:** 10.1111/nph.70815

**Published:** 2025-12-03

**Authors:** Rory Osborne

**Affiliations:** ^1^ School of Biosciences University of Birmingham Edgbaston B15 2TT UK

**Keywords:** H3K27me3, histone demethylation, histone methylation, JmJC, plant stress, PRC2

## Abstract

Subject to an ever‐changing world, plants must respond to harmful conditions and environmental fluctuations. Their evolutionary success can be attributed to their plasticity in both perceiving and integrating these variations to facilitate adaptation. The epigenetic control of gene expression through histone modification affords plants this flexibility by fine‐tuning gene expression and imprinting a transcriptional memory of specific conditions. Histone H3 lysine 27 trimethylation (H3K27me3) is a repressive modification held in balance across the genome by its writer, the Polycomb Repressive Complex 2, and its erasers, Jumonji‐class histone lysine demethylases. While extensively studied as a mark controlling cell‐fate identity and developmental transitions, recent efforts have shown that stress‐responsive loci are also regulated by H3K27me3. In this review, I explore the emerging roles of these H3K27me3 writers and erasers as central hubs in stress adaptation, highlighting their context‐dependent regulation and interplay with other chromatin marks. By examining H3K27me3 dynamics, I provide an updated perspective on its versatile functions beyond development, emphasising its relevance in enhancing plant adaptation and resilience to changing environments.

## Introduction

Eukaryotes organise their genetic material in a complex structure of DNA and protein called chromatin. Approximately 147 bp wrap around protein complexes called nucleosomes, which are comprised of eight histone proteins. Histones possess intrinsically disordered N‐terminal tails that are subject to extensive post‐translational modification, including acetylation, methylation, and ubiquitylation (Luger *et al*., 1997; Kouzarides, 2007). These modifications change chromatin structure by altering the interactions between nucleosomes and the surrounding DNA. While some modifications facilitate a relaxed chromatin conformation that permits RNA polymerase accessibility and gene expression, others such as histone H3 lysine 27 trimethylation (H3K27me3) promote nucleosome compaction, chromatin loop formation, and gene repression.

**Fig. 1:**
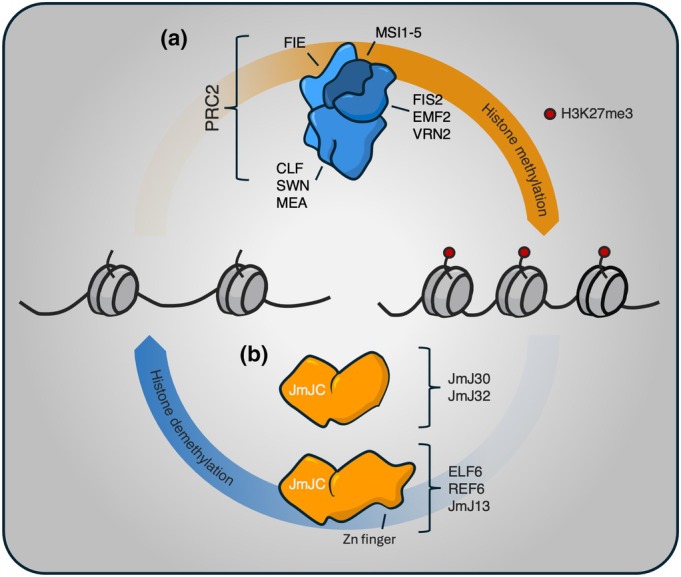
Writers and erasers of H3K27me3. (a) Organisation of the Polycomb Repressive Complex 2 (PRC2) in *Arabidopsis thaliana*. Three subunits are encoded by paralogous homologues, which assemble around the non‐redundant core component FIE. Modular assembly of these homologues gives rise to distinct PRC2s with unique roles in mediating H3K27me3 deposition. (b) Five histone lysine demethylases have so far been reported in Arabidopsis. Each possesses a conserved JmJC domain and can be further characterised by the presence of a Zn finger domain. These proteins share redundant and non‐redundant roles in H3K27me3 removal.

In plants, H3K27me3 is established by the Polycomb Repressive Complex 2 (PRC2). Named for their major orthologues first described in *Drosophila melanogaster*, *Arabidopsis thaliana* (hereby Arabidopsis) PRC2 possesses four major subunits (Fig. 1a); one Extra sex combs scaffold (FERTILISATION‐INDEPENDENT ENDOSPERM, FIE); three Enhancer of zeste (E(z)) histone methyltransferases (MEDEA, MEA; CURLY LEAF, CLF; SWINGER, SWN); three Suppresser of zeste (Su(z)12) nucleosome binding proteins (EMBRYONIC FLOWER 2, EMF2; FERTILISATION‐INDEPENDENT SEED 2, FIS2; VERNALIZATION2, VRN2); and five p55 nucleosomal remodelling factors (MULTICOPY SUPPRESSOR OF IRA; MSI1‐5). In plants, three major isoforms of PRC2 exist based on the Su(z)12 subunits EMF2, FIS2 or VRN2, which provide spatiotemporal and signal‐dependent specificity to the activity of the complex, which is further supported by PRC1‐dependent ubiquitylation of H2A at K119 (Yang *et al*., 2017). The balance of H3K27me3 is maintained by Jumonji‐class (JMJ) histone H3 lysine demethylases (HKDMs), which remove methyl groups at H3K27me3‐marked loci to facilitate chromatin relaxation. So far, five genes with such activity have been described in Arabidopsis: EARLY FLOWERING 6 (ELF6), RELATIVE OF ELF6 (REF6), JMJ13, JMJ30 and JMJ32, which are also conserved across angiosperms (Fig. 1b; Crevillén, 2020).

Both PRC2 and HKDMs have been extensively studied in the context of plant development, with roles spanning the entire plant life cycle (Nottke *et al*., 2009; Mozgova *et al*., 2015). Recent studies, however, have shown that H3K27me3 dynamics also contribute to stress acclimation, environmental memory, and homeostasis. This review summarises these findings and examines the emerging antagonism between H3K27me3 writers and erasers that facilitates adaptation.

## When it rains, it pours: regulation of H3K27me3 during abiotic stress

**Fig. 2:**
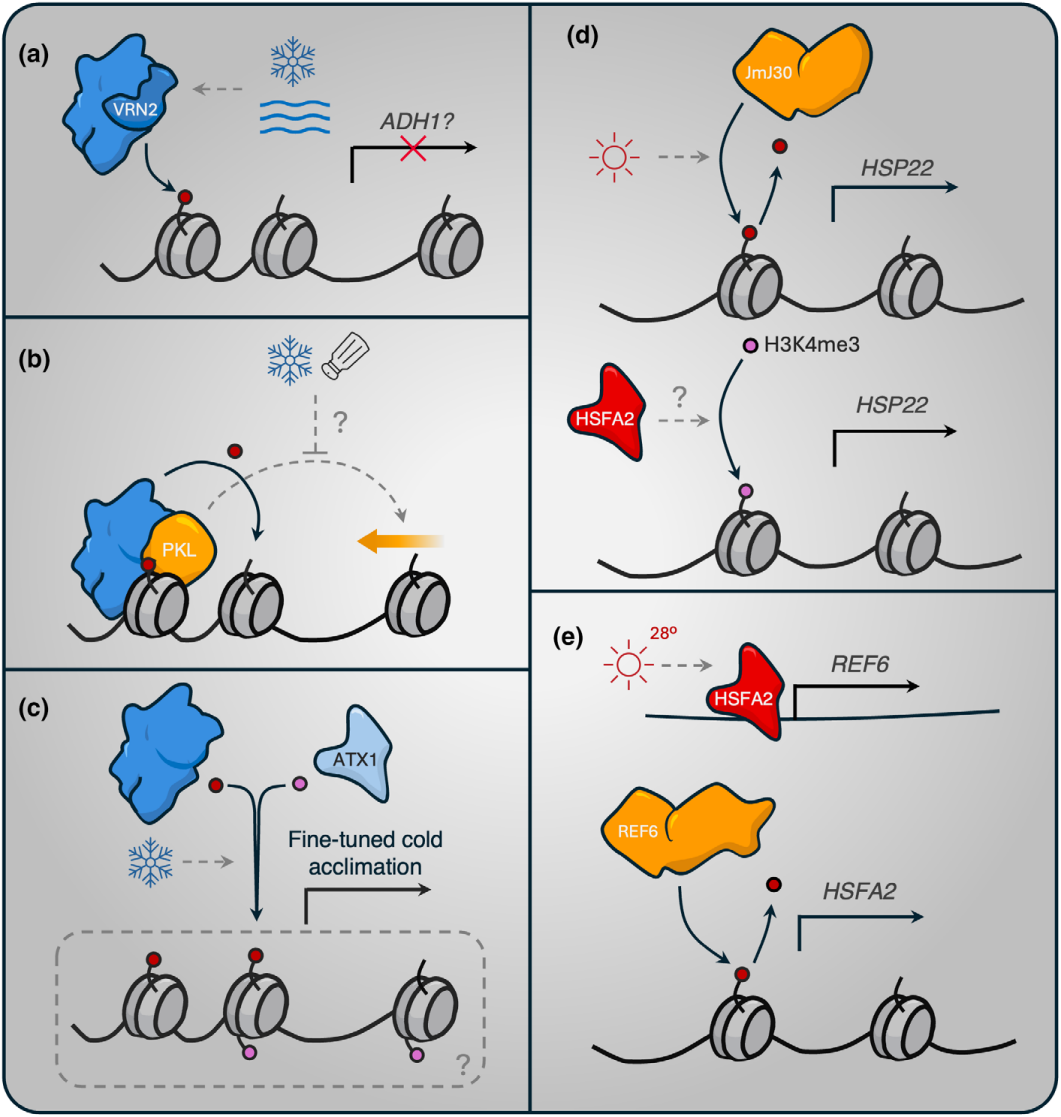
Putative mechanisms of writer and eraser activity in response to abiotic stress. (a) Both cold and flooding stresses stabilise the PRC2 subunit VRN2 by attenuating its proteolytic turnover, implicating it as a regulator of transcriptional networks downstream of these stresses, including the major hypoxia marker *ADH1*. (b) The chromatin remodelling factor PKL compacts nucleosomes at developmental loci to support PRC2‐dependent H3K27me3 spreading from nucleation sites. This function may be conserved in response to abiotic stresses, such as cold and salinity, for which it is already implicated. (c) PRC2 and ATX1 genes fine‐tune cold acclimation through the formation of bivalent chromatin, which is marked with both H3K27me3 and H3K4me3. Whether these modifications exist on the same nucleosome or exist separately across a locus is unclear. (d) High‐temperature‐mediated induction of HSFA2 promotes the expression of *REF6*, which further alleviates H3K27me3 dependent repression of *HSFA2*. (e) JMJ30 coordinates high‐temperature memory by facilitating the removal of H3K27me3 at *HSP21*. This derepression is proposed to increase the accessibility of transactivators such as HSFA2 which indirectly promote H3K4me3 deposition.

The canonical gain of H3K27me3 in response to the abiotic environment is the PRC2‐mediated repression of *FLC* over winter, reviewed extensively (Whittaker & Dean, 2017; Costa & Dean, 2019). This repression requires the isoform VRN2‐PRC2, as well as interacting cofactors and lncRNAs (Tian *et al*., 2019; Fiedler *et al*., 2022; Franco‐Echevarría *et al*., 2023). VRN2 possesses an oxygen‐labile N‐terminus, which subjects it to post‐translational regulation via the N‐degron pathway of protein degradation (Gibbs *et al*., 2011, 2018). While prolonged cold was sufficient to attenuate VRN2 turnover to promote the stability of VRN2‐PRC2, this proteolytic regulation also implicates it in the regulation of hypoxia and flooding responses. Indeed, VRN2 becomes stabilised throughout plant tissues under these conditions, suggesting it acts as a sensor of cold and low O_2_ that influences adaptive PRC2 activity. A potential target is *ALCOHOL DEHYDROGENASE 1* (*ADH1*), which was recently shown to be regulated by H3K27me3 in its promoter (Fig. 2a) (Shimizu *et al*., 2025).

Histone modifications are supported by chromatin remodelling factors, which either extend or shorten the distance between nucleosomes to regulate the propagation of epigenetic marks. The SWI/SNF chromatin remodelling factor PICKLE (PKL), for example, regulates cold acclimation, as well as drought and salt tolerance (Yang *et al*., 2019). The *pkl‐1* mutant was hypersensitive to prolonged cold treatment, which correlated with perturbations to the expression of key genes (*COLD REGULATED 15a*; *COR15A*, *HIGHLY ABA INDUCED 2*; *HAI2*) in the abiotic stress response network. Further investigation revealed that the drought regulator *At‐14‐like1* (*ATFL1*) is also derepressed in *pkl‐1* (Jing *et al*., 2023). *ATFL1* became hypomethylated in the mutant compared to wild‐type seedlings, suggesting PKL represses the drought response by maintaining H3K27me3 at responsive genes. It has since been shown that PKL functions as a major regulator of PRC2 activity and H3K27me3 spreading from nucleation sites at transcriptional start sites (Liang *et al*., 2024). While the binding of PRC2 at target loci was unaffected in *pkl‐1*, the distribution of H3K27me3 across its targets was significantly reduced. Thus, PKL facilitates the condensing of nucleosomes to assist PRC2‐mediated deposition of H3K27me3 across whole loci. Whether PKL associates with adaptive genes during cold or drought conditions to regulate acclimation has not yet been tested (Fig. 2b).

The gain of H3K27me3 at adaptive genes in response to cold was not always associated with transcriptional repression. A study in cold‐treated Arabidopsis seedlings interestingly showed that many such genes also gained the activating histone mark H3K4me3 (H. Wang *et al*., 2024), mediated by the histone methyltransferase ARABIDOPSIS HOMOLOGUE OF TRITHORAX1 (ATX1). Downregulated genes with this bivalent chromatin modification were associated with developmental processes, while upregulated genes were enriched for functions in stress responses, corroborating a similar report in potato tubers (Zeng *et al*., 2019). Compared to wild‐type, the expression of most (*c*. 90%) cold‐responsive genes remained unchanged in *clf‐28* and *atx1‐2* mutants, despite having altered H3K27me3 and H3K4me3 profiles respectively. This points to some redundancy in the regulation of genes through histone bivalency, but nonetheless eludes to a putative regulatory mechanism in a small subset of genes that facilitate cold acclimation (Fig. 2c; Gao *et al*., 2023). While the regulatory role of bivalent chromatin in facilitating long‐term cold adaptation is unclear, it has been shown to support transcriptional memory in response to drought (Liu *et al*., 2014a,b).

A CLF/SWN dependent gain of H3K27me3 has also been observed in response to exogenous ABA treatment in Arabidopsis (Liu *et al*., 2018). This activity targeted senescence genes, where PRC2 is proposed to dampen ABA responses and attenuate hypersensitivity. In support of these findings, the authors conducted a meta‐analysis of transcriptionally induced genes during salt, drought and cold treatments, and evaluated their H3K27me3 status. This revealed an over‐representation of stress‐responsive genes which both gained H3K27me3 and became upregulated, suggesting these may also be regulated through chromatin bivalency.

Other phytohormones have been implicated in coordinating environmental responses through epigenetic regulation. Gibberellic acid (GA) signalling can attenuate PRC2‐mediated deposition of H3K27me3 in rice (Li *et al*., 2023), while reducing endogenous levels of GA promoted thermo‐tolerance through an H3K27me3‐dependent mechanism (Guo *et al*., 2025). Low GA promoted the DELLA‐dependent recruitment of the rice PcG protein LEAF INCLINATION 2 (OsLC2) to attenuate *OsHSFA2d* expression. As GA signalling is an essential component of abiotic stress responses, the authors suggest controlling GA homeostasis is a viable strategy to balance H3K27me3 deposition and enhance heat stress tolerance.

Removal of H3K27me3 has also been described in regulating abiotic stress tolerance, including heat, cold, drought and salinity. For example, in soybean it was shown that the two JmJ‐like histone demethylases *GmJmJ30‐1/2* promote salt stress tolerance by derepressing *GmZF351* (Wei *et al*., 2023). In Arabidopsis, JMJ30, JMJ32, ELF6 and REF6 redundantly prime *HSP17* and *HSP22* by removing H3K27me3 during high temperatures (Yamaguchi & Ito, 2021a,b). A follow‐on study published by the same group showed that removal of H3K27me3 at *HSP* loci correlated with an increase in H3K4me3 that enhanced transcriptional sensitivity (Yamaguchi *et al*., 2021). Although the increase in H3K4me3 was not attributed directly to HKDM activity, the authors concluded that the preservation of H3K27me3 at heat responsive loci in the *jmj* quintuple mutant attenuated H3K4me3 deposition – highlighting the dependence of one mark over another, and the dynamic regulation of specific genes in response to high temperatures (Fig. 2d).

Further work on the high‐temperature response has revealed that REF6 also integrates heat stress memory (Fig. 2e). Treatment of seedlings at 30°C was sufficient to induce expression of *HSFA2*, and promote HSFA2 binding to a heat shock element in the *REF6* promoter (Liu *et al*., 2019). Increased REF6 levels under high temperatures reduced H3K27me3 at the *HSFA2* locus, forming a positive feedback loop that supported transgenerational heat shock memory by relieving *HSFA2* repression in subsequent progeny.

PRC2 and HKDMs possess extensive roles in modulating abiotic stress responses in plants. In most cases, our understanding of this regulation is descriptive, lacking a complete mechanism explaining how these complexes are targeted to specific genes under different conditions. Most abiotic stresses are not acute and are perceived gradually over time, potentially highlighting phytohormone signalling as a regulator of their activity. This is especially true of PRC2, which has a recurring role in dampening downstream responses, that may subsequently influence its own activity.

## Remember thy enemy: H3K27me3 dependent regulation of biotic stress responses

Our understanding of chromatin dynamics during plant‐microbe interactions has substantially improved in recent years. While this field has focused on DNA methylation as a major target for stable and heritable defence priming (Dowen *et al*., 2012; Atighi *et al*., 2020; Annacondia *et al*., 2021), the significance of histone modifications during biotic stress is becoming increasingly recognised (Baum *et al*., 2019; Ding *et al*., 2021; Nobori *et al*., 2025).

The role of H3K27me3 deposition in response to infection has been well studied. The PRC2 methyltransferase *MEA* was induced during infection of Arabidopsis with *Pseudomonas syringae pv Tomato DC3000* (*Pst*) expressing the effector *AvrRpt2* (Roy *et al*., 2018). Mutant *mea‐6* plants were more resistant to infection and mounted a stronger response to infection compared to Col‐0. Interestingly, a concurrent reduction in H3K27me3 and gain of H3K4me3 across the *MEA* locus during infection suggests PRC2 attenuates the defence response to avoid autoimmunity. A negative regulatory role for CLF and SWN in regulating effector‐triggered cell death has also been described. Inducible expression of *AvrRpt2* or *Necrosis‐Inducing Phytophthora Protein 1* (*NiPP1*) induced the loss and gain of H3K27me3 at hundreds of genes, respectively (Dvořák Tomaštíková *et al*., 2021). While loci gaining the mark were over‐represented by *NAC* and *WRKY* transcription factors and genes involved in ABA signalling, those losing H3K27me3 were predominantly linked to ethylene signalling. Although confirmation with an EZH2 mutant was not directly tested, a meta‐analysis revealed that many genes gaining H3K27me3 overlapped with previously reported CLF/SWN targets, suggesting that PRC2 might repress the onset of effector‐triggered cell death by dampening ABA and senescence signalling.

**Fig. 3:**
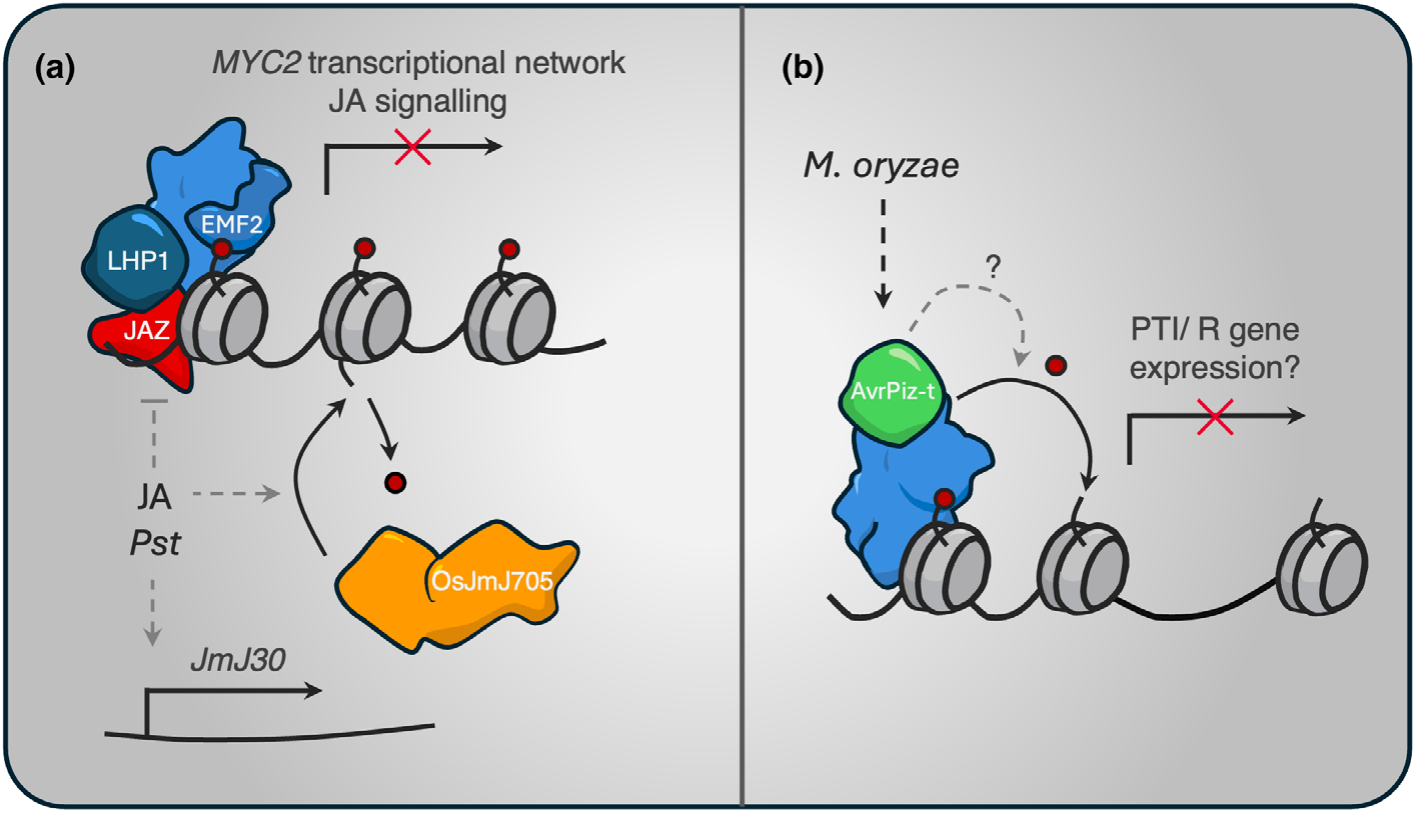
Biotic stress responses are regulated by H3K27me3 dynamics. (a) EFM2‐PRC2 requires LHP1 and JAZ‐domain proteins to suppress jasmonic acid signalling. An increase in JA levels promotes the degradation of JAZ proteins, releasing LHP1 and the repressive activity of PRC2 to promote JA responses and the MYC2 transcriptional network. In Arabidopsis, JmJ30 expression is induced during challenge by the hemi‐biotroph *Pseudomonas syringae pv Tomato DC3000*, while in rice, OsJMJ705 directly demethylates JA signalling genes to promote JA responses. (b) The effector protein AvrPiz‐t secreted by *Magnaporthe oryzae* directly interacts with PRC2 and promotes its activity *in vitro*. Whether this function is conserved *in planta* to attenuate PTI and promote virulence remains unknown.

PRC2 also appears to regulate jasmonic acid signalling. A methyl‐jasmonic acid‐dependent gain of H3K27me3 was overwhelmingly associated with genes involved in auxin signalling, glucosinolate production and lipid localisation (Li *et al*., 2021; Vincent *et al*., 2022). Parallel mapping of the activating H4ac mark revealed a colocalisation with H3K27me3 in immune response genes, including TIR‐NLR class resistance genes. Furthermore, EMF2 and LHP1 have been shown to interact with JAZ‐domain proteins. The degradation of JAZ proteins in response to Me‐JA was sufficient to release PRC2, and alleviate the repression of anther development. In support of the developmental role of H3K27me3 in regulating immunity, mutation of the LHP1 promoted the derepression of genes in the JA/ethylene branch of defence signalling (Ramirez‐Prado *et al*., 2019). The same study also showed that the *lhp1* mutant had reduced levels of salicylic acid, the biosynthesis of which was further impaired during *Pst* infection. This phenotype was attributed to the H3K27me3‐dependent regulation of the transcriptional network downstream of the master regulator MYC2 (Fig. 3a).

The role of PRC2 in balancing defence‐hormone signalling has also been highlighted through its regulation of the lncRNA *SALICYLIC ACID BIOGENESIS CONTROLLER 1* (*SABC1*) (Liu *et al*., 2022). CLF directly interacts with *SABC1*, which promotes the association of PRC2 with the overlapping gene *NAC3*, which is constitutively downregulated in unstressed conditions. The downregulation of *SABC1* during pathogen infection subsequently reduces PRC2 association and H3K27me3 levels at *NAC3*, facilitating its derepression and promotion of salicylic acid biosynthesis during both *Pst* and *TuMV* challenges. In the absence of an associated HKDM, it is unclear whether the maintenance of PRC2 at the *SABC1* locus is essential for nucleosome compaction, or whether an additional factor is subsequently recruited at *NAC3* to promote expression during biotic stress. Interestingly, *SABC1* repression of *NAC3* requires chromatin loop formation, implicating PRC2 accessory proteins as mediators of this regulation.

Evidence also suggests that PRC2 is directly targeted by pathogens to suppress immunity. A study in rice has shown that *Magnaporthe oryzae* infection increases global levels of H3K27me3 (Zheng *et al*., 2024). Interestingly, *M. oryzae* utilises the effector AvrPiz‐t to enhance PRC2 activity. AvrPiz‐t interacted with the Polycomb protein OsSDG711, which promoted its methyltransferase activity *in vitro* (Fig. 3b). As a regulator of both development and stress responses, the direct modulation of H3K27me3 deposition by a pathogen has remarkable implications for our understanding of how microbes have evolved to suppress immunity, and is likely a strategy utilised by other organisms. It has also been shown that H3K27me3 is deposited at a conserved late element (*CLE*) in tomato golden mosaic (TGMV) and cabbage leaf curl viruses (CaLCuV) (Williams *et al*., 2024). *cle‐* variants of both TGMV and CaLCuV were less virulent in Arabidopsis, correlating with the reduced expression of coat protein gene expression, which is driven by *CLE*. Binding by the host transcription factor TEOSINTE BRANCHED 24 (TCP24) to *CLE* was proposed to influence histone modification across the viral genome by recruiting PRC2, although no interaction between TCP24 and PRC2 was demonstrated. Whether H3K27me3 deposition at this region is part of the plant's molecular defence to silence viral transcription or has become exploited as an infection strategy by Geminiviruses to protect their genome integrity, centred around the vital *CLE* region, warrants further investigation.

While the gain of H3K27me3 during plant‐microbe interactions has been well characterised, there is currently less evidence for an adaptive role of HKDMs in regulating disease responses. In rice, *OsJmJ705* was shown to enhance resistance to *X. oryzae* by derepressing JA‐induced gene expression (Li *et al*., 2013). Furthermore, *JmJ30* was induced during the early stages of *Pst* infection in Arabidopsis (Fig. 3a; Yamaguchi & Ito, 2021a,b). Interestingly, REF6‐dependent thermomemory came at the cost of pathogen resistance, with the derepression of *HSFA2* in response to heat leading to reduced SA biosynthesis during *Pst AvrRPT2* infection (Liu *et al*., 2019). This underscores an important caveat to stress memory, in that priming against one stimulus is likely to impact the response to another. This is especially true for abiotic vs biotic stresses, which broadly utilise distinct mechanisms to facilitate adaptation. Nonetheless, given the prevalent role of H3K27me3 erasers in abiotic responses, it is likely that these play a considerable role in defence responses, representing an untapped avenue for improving our understanding of plant disease resistance.

## Balancing act: maintaining homeostasis in fluctuating conditions through H3K27me3 dynamics

Natural variations in the abiotic environment occur throughout the day and across the seasons. These include the day/night cycle, changes to light quality, shifts in ambient temperature and nutrient availability. While not considered stressful *per se*, plants must still respond to these changes to maintain homeostasis.

In *Arabidopsis helleri*, it was shown that developmental plasticity is mediated through oscillating H3K27me3 and H3K4me3 throughout the day and over the year (Nishio *et al*., 2020). H3K27me3 levels were stable throughout the day/night cycle, but fluctuated across the seasons. While accumulation of H3K27me3 at *FLC* during the colder months was unsurprising, other genes, including *AhgLTI30* and circadian clock genes such as *AhgTOC1*, also experienced seasonal gains and losses of the mark. Reduction of the modification at many loci was also associated with enrichment of H3K4me3, implying regulation by both marks at adaptive genes throughout the year. Interestingly, these oscillatory patterns appeared synchronously at genes regulating temperature responses, suggesting a role for bivalency in seasonal temperature acclimation (Nishio *et al*., 2020).

Both warm temperature and light responses are regulated through the photoreceptor phytochrome B (phyB). In brief, red light induces structural changes that activate phyB, promoting its translocation to the nucleus where it binds to PHYTOCHROME INTERACTING FACTOR1‐8 (PIF1‐8) transcription factors (Lorrain *et al*., 2008; Kaiserli *et al*., 2015). The sequestration of PIFs by phyB facilitates their phosphorylation and degradation. Shade conditions (far‐red) revert phyB to its inactive form, allowing PIFs to accumulate and reprogram gene expression to promote the shade avoidance response. As ambient temperatures increase, the inactivation of phyB is accelerated, promoting PIF activity (Legris *et al*., 2016). Both shade avoidance and thermomorphogenesis are influenced by H3K27me3 dynamics within the PIF transcriptional network.

PKL, for instance, enhances the induction of the PIF4 targets, *IAA19* and *IAA29*, in response to warm ambient temperature, by indirectly facilitating the removal of H3K27me3 (Zha *et al*., 2017). Both genes, which promote cell expansion, became hypermethylated in a *pkl* mutant, which correlated with their reduced expression. While the authors did not investigate any responsible HKDM, a likely candidate is REF6, which has been implicated in the imprinting of shade and thermomemory (He *et al*., 2022) and interacts with PIF4 (Yan *et al*., 2025). REF6 regulates the expression of *GA20ox2* and *bHLH87*, two downstream targets of PIF4. While the warm temperature induction of *GA20ox2* and *bHLH87* was not dependent on the removal of H3K27me3 at these loci, their hypermethylation was observed in the non‐functional *ref6‐5* mutant. This correlated with a dampening of their responsiveness during the transition from 22°C to 28°C, suggesting that REF6 is required to keep these genes in a primed state. The establishment and maintenance of this primed state have been further highlighted in the shade memory response. Synergistic activity between REF6/ELF6 and PIF7 at shade avoidance genes, including *IAA19*, suggests that H3K27me3 removal contributes to an epigenetic memory of shade that potentiates the shade avoidance response (Burko *et al*., 2022; Cheng *et al*., 2024).

While HKDMs appear to promote PIF/phyB signalling, emerging evidence suggests this activity is directly antagonised by PRC2. The Polycomb protein VERNALIZATION INSENSITIVE 2–LIKE1/VERNALIZATION5 (VIL1) was reported to inhibit plant growth by repressing the PIF4 target *HAT4* in the light (Kim *et al*., 2021). While H3K27me3 levels across *HAT4* remained constant throughout the day, the association of VIL1 at the *HAT4* locus peaked at midday, correlating with its recruitment of active phyB. PRC2‐dependent maintenance of H3K27me3 required both VIL1 and phyB, which formed a repressive chromatin loop within the *HAT4* promoter to attenuate its ectopic expression in the light. Light‐dependent degradation of VIL1 through the phyB antagonist, CONSTITUTIVELY PHOTOMORPHOGENIC 1, highlights the role of H3K27me3 deposition in fine‐tuning gene expression in response to fluctuating environmental signals (W. Wang *et al*., 2024). A follow‐on study by the same group revealed that VIL1 also negatively regulates thermomorphogenesis, demonstrated by the failure to deposit H3K27me3 at warm temperature‐responsive loci in the *vil1‐1* mutant (Kim *et al*., 2023). Interestingly, VIL1 also indirectly influences H2A.Z eviction, potentially through the misregulation of its H3K27me3 targets. Chromatin bivalency has also been identified as contributing to warm temperature acclimation through LHP1 and ethylene signalling (Shao *et al*., 2024).

Corroborating the observation that VIL1 regulates light‐mediated growth, it was also shown that VRN2‐PRC2, which physically interacts with VIL1, facilitates H3K27me3 deposition at key PIF4 target genes during early leaf emergence (Osborne *et al*., 2025). Mitotically stable H3K27me3 deposited by stabilised VRN2 in the hypoxic shoot meristem is maintained during leaf expansion and is required for the VIL1/phyB‐dependent loop in the *HAT4* promoter. Taken together these studies highlight a role for PRC2 in regulating growth by restricting ectopic expression of PIF4 target genes in the light. While REF6 and PRC2 both regulate light and temperature responses, direct antagonism between them at a mechanistic level has so far not been described. Their respective roles in regulating these physiological responses likely converge on PIF4 and phyB through an unknown mechanism (Fig. 5a, see later).

PRC2 also modulates adaptation to nutrient and energy availability. The Polycomb protein FIE, which is the only constitutive subunit of all plant PRC2s, was recently identified as a phosphorylation target of TOR kinase (Ye *et al*., 2022). Inhibition of TOR reduced levels of H3K27me3 across the genome, while H3K4me3 and H3K9me2 levels remained unchanged. Increased glucose availability enhanced TOR‐dependent phosphorylation of FIE, inducing its translocation from the cytoplasm to the nucleus. This translocation potentiated the vernalisation response (Fig. 5b, see later), suggesting FIE phosphorylation acts as a key checkpoint in the activity of PRC2 in regulating the floral transition in response to cold.

Whether the stress‐induced energy crisis is the primary driver of adaptive PRC2 activity in the context of environmental responses is still unexplored. Indeed, stress‐responsive genes, which are induced by TOR inhibition, are regulated through bivalent chromatin formation dependent on H3K27me3 and H3K4me3 deposition by CLF/LHP1 and an unknown histone methyltransferase (Fig. 5b, see later) (Dong *et al*., 2023). This activity was directly antagonised by the chromatin remodelling protein BRAHMA, which has an opposing function to PKL in regulating H3K27me3 deposition and maintenance.

Energy homeostasis is concurrently regulated by the removal of H3K27me3 by HKDMs. The major energy‐sensing protein SNF1‐related kinase (SNRK1), considered an antagonist to TOR activity (Margalha *et al*., 2019), was shown to promote H3K27me3 removal through JMJ705 in rice (Wang *et al*., 2021). Mutation of the *JmJ705* coding sequence severely attenuated seedling tolerance to prolonged starvation, which was linked to the dampening of genes linked to energy mobilisation. Phosphorylation of JmJ705 by SNRK1 subsequently promoted demethylase activity, which largely targeted genes with functions in the starvation response (Fig. 5b, see later).

Thus, while developmental transition appears to be gated through energy‐status mediated by PRC2 and TOR, the JmJ‐SNRK1 regulatory module potentiates starvation responses. Whether the dynamics of H3K27me3 deposition within these contexts operate independently, antagonistically, or target overlapping gene networks remains unclear.

## Discussion

Maintaining homeostasis requires the continual surveillance and integration of environmental signals that drive physiological responses. The transcriptional programmes induced by stress must be fast, efficient and robust, yet readily switched off to avoid unnecessary energy expenditure and the negative impacts of hyperactivity. As sessile organisms, plants must be flexible in how they adapt to the environment, and histone modifications help facilitate this plasticity. This is especially true in instances where H3K27me3 colocalises with activating marks such as H3K4me3 or H4ac.

Bivalent chromatin was first described in 2006 (Azuara *et al*., 2006; Bernstein *et al*., 2006), and shortly after in Arabidopsis in 2010 (Deal & Henikoff, 2010). Since then, reports of genes possessing multiple chromatin states to facilitate long‐term adaptive responses to stress are well documented (Faivre & Schubert, 2023). Whether bivalency configures a poised, permissive, or repressive state in plants remains an open question. The emergence of new methods such as ReChIP (Desvoyes *et al*., 2018), single‐cell ChIP (Feng & Mann, 2022) and chromatin accessibility profiling (Grandi *et al*., 2022) will be especially useful in deciphering the structure and function of these nucleosomal states, especially in complex tissues where individual cells are likely to possess distinct epigenetic profiles.

**Fig. 4:**
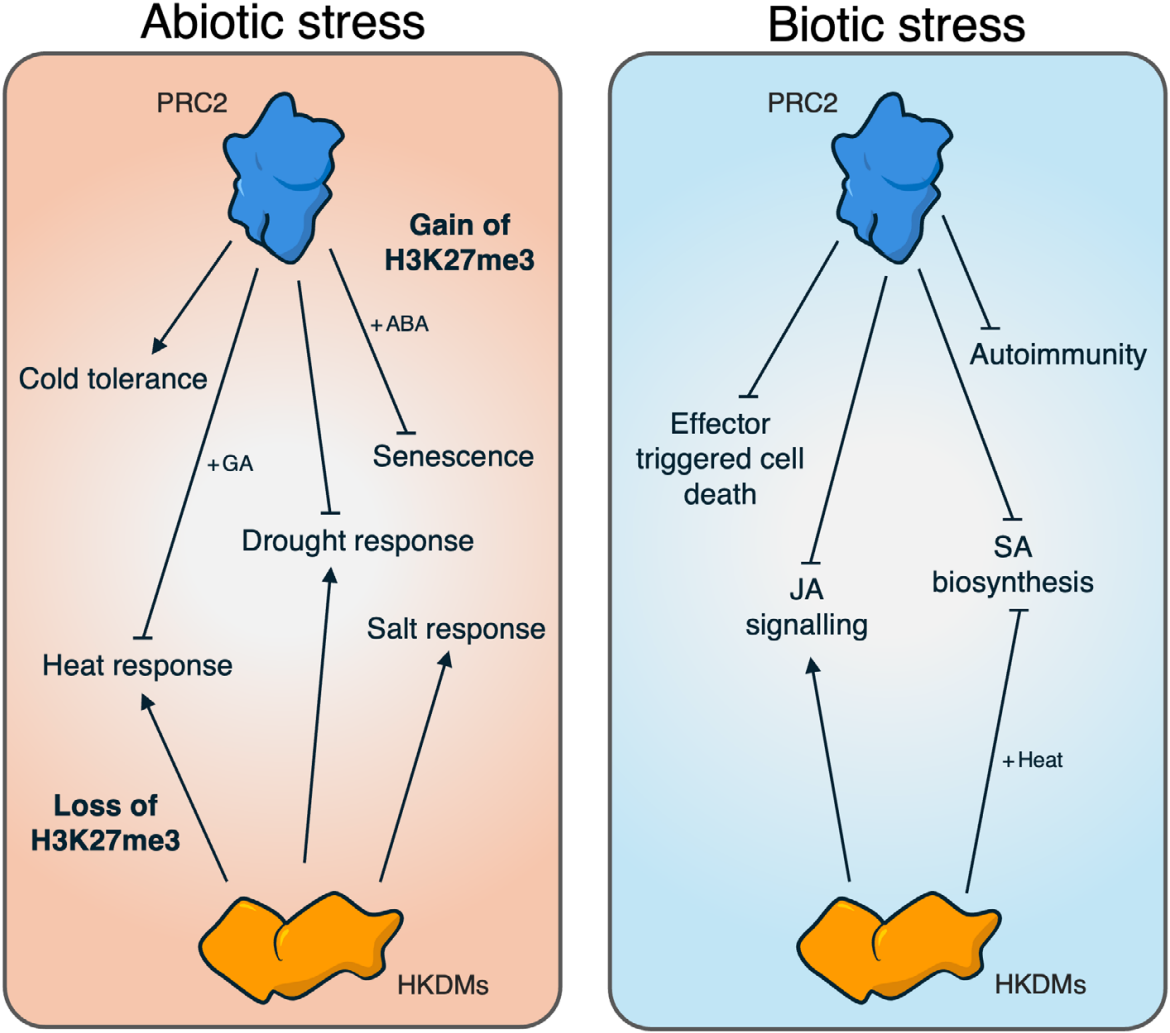
Distinct and antagonistic activities of PRC2 and HKDMs in stress adaptation. Pointed and blunt arrows indicate positive and negative regulation of biological processes respectively.

**Fig. 5:**
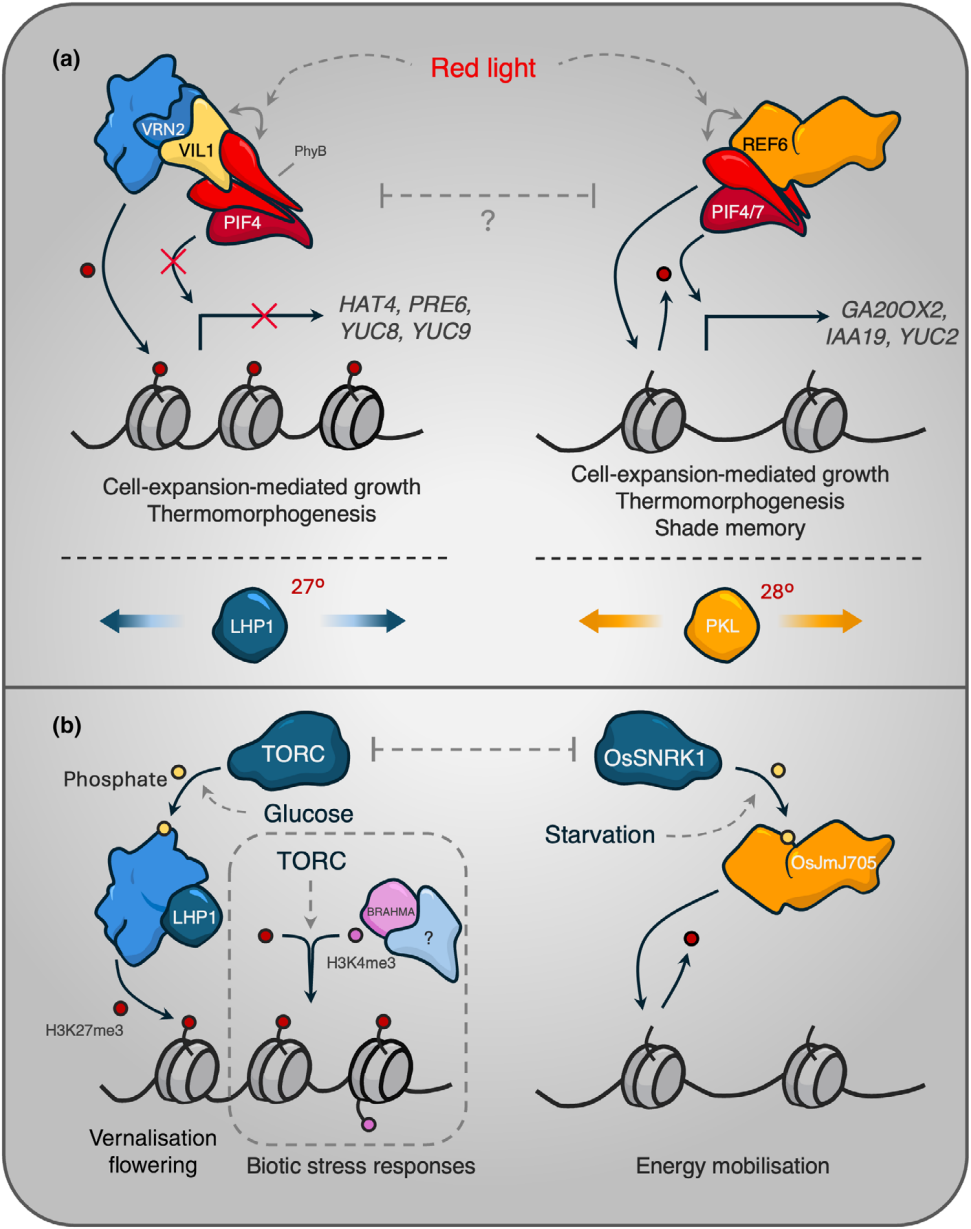
Antagonism between H3K27me3 writers and erasers fine‐tunes homeostasis in fluctuating conditions. (a) Red light promotes the interactions between VIL1‐phyB and REF6‐phyB, respectively. Whether this interaction is competitive between components of the H3K27me3 regulatory machinery is unclear. These interactions target both PRC2 and REF6 to regulate distinct targets as part of the shade avoidance response, growth, and warm‐temperature acclimation. Both PKL and LHP1 have been shown to positively regulate the ambient warm temperature response by promoting nucleosome relaxation and chromatin bivalency, respectively. (b) The energy‐status antagonists TORC and SNRK1, respectively, phosphorylate PRC2 and OsJMJ705 in response to fluctuating energy availability. Phosphorylated FIE/PRC2 mobilises to the nucleus, where it represses genes involved in vernalisation. The repressive activity of TOR/PRC2/LHP1 also converges on bivalent genes marked with H3K4me3 by an unknown H3K4me3 methyltransferase that requires BRAHMA. Whether the activity of TORC and SNRK1 gates adaptive responses warrants further investigation. Solid black arrows indicate post‐translational modifications; solid grey arrows indicate physical interactions; dashed grey arrows indicate positive regulation by specified factors, while blunt ended grey arrows indicate negative repression.

The spatiotemporal impact of histone modifications on chromatin structure also remains unclear. Epigenetic stress memory is clearly an essential component of plant adaptation, but how long does this memory last, and what controls the decision to maintain or erase specific histone marks in favour of others? The repression of *FLC* is maintained after vernalisation until the next generation, where it is reset during embryogenesis (Sheldon *et al*., 2008). Are other H3K27me3 targets regulated in the same way, or are memories written and erased through antagonistic activities of different histone‐modifying complexes? PRC2 and HKDMs seem to play opposing roles during stress adaptation (Fig. 4), converging on biological processes albeit through distinct gene targets. This is especially true of thermomorphogenesis, where both PRC2 and REF6 require PIFs to regulate thermomemory and growth (Fig. 5a). Direct antagonism between PRC2 and a JmJC demethylase has been described in murine stem cells (Shen *et al*., 2009), wherein the latter binds and attenuates the methyltransferase activity of mammalian PRC2. Decoding any direct relationships between plant PRC2s and HKDMs will improve our understanding of how epigenetic memory is maintained, which is especially important given that this phenomenon has likely evolved to accommodate environmental predictability.

A hallmark of rising CO_2_ levels is intense fluctuations in climate between and within seasons, presenting a unique challenge to both somatic and transgenerational adaptation. Most studies have so far focused on short‐term memory in Arabidopsis. How do H3K27me3 dynamics facilitate long‐term adaptation in crops, or even perennial plant species? PRC2 and its individual components are ancient and conserved across flowering plants. In contrast to animal models, genes encoding plant PRC2 subunits have also experienced multiple duplication events across green lineages, giving rise to different homologues with distinct functions. This suggests that while the role of PRC2 in imprinting adaptive memory is conserved, its gene targets are not. This is also true for HKDMs, which appear across the angiosperms, but likely possess distinct roles and functions (Huang *et al*., 2016).

Most HKDMs directly bind DNA through a conserved zinc‐finger domain, whereas PRC2 appears not to directly bind DNA. While a Polycomb Responsive Element has been described (Xiao *et al*., 2017), many genes repressed by PRC2 do not possess such elements. Instead, PRC2 relies on cofactors to provide target specificity in response to distinct environmental stimuli (Godwin & Farrona, 2022). This permits fluidity in its activity but presents a challenge in describing PRC2 dynamics during different conditions. For example, both VIL1 and VRN2 have been reported to regulate PIF4 target genes, but neither protein interacts with PIF4. Understanding the role of PRC2 cofactors in different environmental contexts will be critical in explaining H3K27me3 dynamics during them. This is especially true for chromatin remodellers like PKL, and H3K27me3 readers such as LHP1, which can act both positively and negatively with respect to PRC2 activity. Finally, how PRC2 and HKDMs perceive environmental fluctuations warrants further investigation. What signals do writers/erasers receive to indicate specific loci are destined for modification? This also likely depends on the activity of accessory proteins, which influence writer/eraser activity in a context‐dependent manner (Khan *et al*., 2025). The nutrient‐dependent phosphorylation of both enzymes is also a possible factor, as well as environmental and phytohormone‐dependent regulation of Polycomb subunits, cofactors and interactors. Other mechanisms may also signal to histone modification machinery, such as organellar stress signalling or direct activation through plasma membrane‐mediated signalling cascades.

## Conclusions

Chromatin remodelling allows plants to fine‐tune gene expression in response to the biotic and abiotic world. This is achieved through the dampening or activation of downstream transcriptional networks. While significant advances in our understanding of these dynamics have been made in recent years, open questions have emerged which require further attention (Box 1).

Box 1 **Open questions**

- How are H3K27me3 writers/erasers targeted to specific loci under different conditions? Do these enzymes perceive stress directly, or do accessory proteins act as primary sensors that transmit environmental signals to drive histone modification?
- How do different/multiple chromatin marks work together to create an epigenetic landscape that more finely regulates gene expression?
- What is the spatiotemporal impact of epigenetic marks on transcriptional memory? How long do these memories last, and in which tissues are they held?

The continued investigation into the activity of proteins which regulate H3K27me3 across the genome will help improve our understanding of stress adaptation and how plants have evolved to thrive in an ever‐changing environment.

## Competing interests

None declared.

## Disclaimer

The New Phytologist Foundation remains neutral with regard to jurisdictional claims in maps and in any institutional affiliations.
